# Improving the Reliability and Utility of Streptozotocin-Induced Rat Diabetic Model

**DOI:** 10.1155/2018/8054073

**Published:** 2018-09-23

**Authors:** Yanlin Wang-Fischer, Tina Garyantes

**Affiliations:** Department of Therapeutics, Chromocell Corporation, 685 U.S. Highway One, North Brunswick, NJ 08902, USA

## Abstract

The Streptozotocin- (STZ-) induced diabetic model is widely used; however, unexplained acute toxicity has given the model an unreliable reputation. To improve the reliability and utility of this model, we characterize the age dependence of STZ toxicity and introduce novel endpoints to assess diabetic complications and reveal possible mechanisms for diabetic development. Diabetes was induced by STZ injection into male, 6 to 23 weeks old, Sprague-Dawley rats. Their metabolic (glucose, lipids, and hormones), inflammatory (cytokines), histologic and behavioral endpoints were observed for 1.2 years. Analgesic compounds were assessed for efficacy treating neuropathy. Acute mortality, within a week of STZ injection (50–65 mg/kg i.v.), was inversely correlated to animal age. Only 3% of rats, age 6–11 weeks, died in the week following STZ injection, whereas 83% of rats 12 to 17 weeks old and 91% of rats 18 weeks or older died in the same week. Partial model recovery (normalized insulin, glucose and food/water intake) was observed starting at week 36; however, pain scores, kidney enlargement, and cataract formation continued to show progression consistent with the diabetic state. Unique noninvasive observational measurements, such as haircoat quality and diarrhea scores, served as useful endpoints for this model. The increased plasma cytokines (such as TNF-*α*, IL-4, and IL-6) and inflammatory cell infiltration into the pancreatic islets are strong evidence of inflammation in the STZ-induced diabetic model. Pancreatic tissue staining revealed total islet area reduction and confirmed STZ-specific pancreatic toxicity; however, the *β*-cell density per area in pancreatic islets and insulin levels statistically increased over time in the diabetic rats, suggesting a mechanism for partial recovery of diabetic symptoms. Voltage-gated sodium channel (NaV1.7 specific, peripherally restricted) blocker, CC4148, inhibited neuropathy without side effects as compared to a nonspecific sodium channel inhibitor, Mexiletine, or GABA analog, Pregabalin, which inhibited neuropathy with side effects.

## 1. Introduction

Diabetes is generally characterized as type 1, the body not producing enough insulin due to a loss of the insulin-producing *β*-cells in the pancreas islets, or type 2, where fat, liver, and skeletal muscle cells do not respond to insulin leading to a reduction in their uptake of sugars from the blood stream [1]. Although most patients are type 2 diabetics because hyperglycemia is the principal factor in the etiology and pathogenesis of diabetic complications, both type 1 and type 2 diabetic models are used to study diabetic complications and their potential treatment [1–3]. Type 1 diabetic models are usually easier and cheaper to establish and, therefore, are employed whenever possible. The most common animal model of type 1 diabetes, using STZ intraperitoneal or intravenous (i.p. or i.v.) injection, has been in extensive use since 1963 [1, 2, 4, 5]. STZ is a naturally occurring chemical, a broad-spectrum antibiotic that is particularly toxic to the insulin-producing *β*-cells of the pancreas. Because of this selective toxicity, it is also used to treat *β*-cell pancreatic cancers [4, 5]. According to a review, “Despite the large number of publications on the topic, more than 17,000 listings for Streptozotocin on PubMed, investigators inexperienced with a model of STZ-induced diabetes may find it difficult to precisely design new studies. STZ-induced diabetes can be highly variable, and no standard protocol exists for the preparation, dose, or administration of STZ” [6]. Most papers include only the animals where diabetes was successfully induced and provide very little technical details regarding failures during diabetic induction, long-term mortality, and severity of diabetes experienced. However, this information is important for successful diabetic model induction and by improving reproducibility and utility of the model can benefit animal welfare by reducing acute animal mortality and morbidity. Since diabetes in humans is a lifelong chronic disease, long-term observation of this chronic model and its natural recovery should aid the study of diabetes. This report examines the age dependence of acute STZ toxicity and describes the long-term natural course of the model including glucose, HbA1C, lipid, metabolic hormones (such as insulin, glucagon), cytokine levels and clinical conditions, mortality, natural recovery, and multiple diabetic complications resulting from the end-stage of uncontrolled diabetes including the description of novel, stress-free, reproducible behavioral endpoints. The report also includes important information regarding histological changes in *β*-cells of the pancreatic islets and inflammatory cell infiltration into pancreatic tissue confirming diabetic damage and the involvement of inflammatory mediators in diabetic mechanisms. It is well documented that inflammation mediators and oxidative stress play a big role in the pathogenesis and prevalence of both type 1 and 2 diabetes mellitus [7–12]. Since nerve damage from diabetic hyperglycemia causes pain, several existing and potential pain drugs and Chromocell novel NaV1.7 blocker compound were evaluated as pain treatments in the subject animals.

## 2. Materials and Methods

### 2.1. Subjects

Sprague-Dawley male rats (Crl: SD) were originally sourced from Charles River Labs Inc. and bred at Rutgers University. The rats were 6 to 23 weeks old at the beginning of the study. Two animals were housed per cage, at 20–22°C with a 12-hour light/dark cycle. Filtered tap water and rat chow pellets were available ad libitum. Soft gel food (25–50 g/dish/day/rat, Diet Gel 76A) was provided to rats exhibiting severe dehydration or otherwise recommended for extra care by the Rutgers' veterinarian. Cage bedding was changed frequently following the induction of diabetes due to polyuria. Animals were maintained without antihyperglycemic treatment (insulin). A total of 153 rats were included in this study; 130 rats received STZ injection to induce diabetes, and the remainder were injected with 0.01 M sodium citrate as nondiabetic controls (sham). All injections were 2 ml/kg i.v. The STZ-injected rats were grouped by body weight; group 1 included 67 rats 220–400 g (6–11 weeks old); group 2 included 29 rats 401–500 g (12–17 weeks old); and group 3 included 34 rats 501–600 g (18–23 weeks old). STZ injection regimens are described in Table 1.

About 100 *μ*l blood was collected weekly or biweekly during the observation period and used to measure blood glucose and lipid levels, and 100 *μ*l plasma was collected (in K2-EDTA) and stored at −80°C for analysis of cytokine and metabolic hormone levels as shown in the results.

All animal care and experimental procedures were performed in an AAALAC-accredited facility under a Rutgers IACUC-approved animal care and use protocol in accordance with the Guide for the Care and Use of Laboratory Animal.

### 2.2. Drugs and Reagents

STZ (VWR, Cat# 97063-180) was dissolved in 0.01 M sodium citrate (Sigma-Aldrich, Cat# W302600), pH of 4.5 [4, 6]. Although STZ prepared in saline solution is reported by other researchers, in a pilot study, we observed bubbles in the solution and were concerned that the bubbles may affect STZ activity or stability. A study by Al-Hariri [13] had previously shown that the type of solvent used to dissolve and administer STZ affected the success rate of diabetic induction, and diabetes developed faster in rats where STZ was dissolved in distilled water compared to normal saline. We also found STZ dissolved more rapidly in 0.01 M sodium citrate than in water.

(S)-Pregabalin (eNovation Chemicals, Cat#: D320170) was dissolved in drinking water. Mexiletine HCl (VWR, cat# M2040) was dissolved in normal saline (0.9%NaHCl), pH 7.

CC4148 is a peripherally restricted voltage-gated sodium channel (NaV1.7) inhibitor synthesized by Chromocell Corporation. It had previously shown pain inhibition in a rat formalin pain model. For each experiment, an appropriate amount was dissolved in 100% polyethylene glycol 600 (PEG600, Fisher Scientific, Cat# 50-014-45368), with constant stirring on an 80–90°C hotplate, and then diluted in water to a final solvent concentration of 20% PEG600/80% water. Prior to use, the PEG600 was warmed at 37°C due to its gel-like consistency at room temperature (22°C).

### 2.3. Animal Model Establishment

Type 1 diabetes was induced in group 1 (body weight 220–400 g) by either a single i.v. injection of STZ at 65 mg/kg or by two i.v. injections of STZ at 50 mg/kg 3 days apart; group 2 (body weight 401–500 g) received a single dose of STZ at 65 mg/kg; group 3 received either a single dose of STZ at 65 mg/kg or at 50 mg/kg, see Table 1. STZ was freshly dissolved in 0.01 M sodium citrate and dosed in a volume of 2 ml/kg i.v. injection via the tail vein. Sham rats received only the vehicle (0.01 M sodium citrate) in a volume of 2 ml/kg i.v. via the tail vein. Three days after injection, each animal's diabetic status was confirmed by measuring blood glucose levels from tail tip samples taken under nonfasted condition. The glucose level was analyzed using the AlphaTRAK 2 mini glucose meter (Abbott Laboratories USA, cat# 32107-01). Blood glucose was tested between 10 am and 12 pm. Only rats with a blood glucose level ≥ 400 mg/dl were included in the study for diabetic neuropathy. All sham animals showed normal glucose levels. Glycated hemoglobin (HbA1C) was tested at week 11 and week 19 after a STZ injection using an HbA1C Monitor (Wilburn Medical USA, Cat# 3021).

Diabetic rats, surviving acute STZ toxicity, were randomized into treatment groups. Drug treatments for STZ-induced diabetic neuropathic pain included Pregabalin, Mexiletine, and CC4148. The vehicles were water, saline, and 20%PEG600/water for Pregabalin, Mexiletine, and CC4148, respectively; a pilot study showed there was no effect or difference between these 3 vehicles; therefore, only the vehicle for CC4148 was included this study, Table 2.

### 2.4. Assays for Blood Lipid Profile, Plasma Metabolic Hormones, and Cytokines

Blood lipid profile was tested at weeks 11, 15, 19, and 23 after a STZ injection between 10 am and 12 pm after a 4–6 hour fasting period, using an LDX Analyzer (Cat# 13-454, Lipid cassette Cat# 10-989) from Wilburn Medical USA.

Plasma samples for cytokine tests were collected at weeks 2, 4, and 9; plasma samples for hormone tests were collected at weeks 20 and 25 after a STZ injection. Plasma was stored at −80°C until analysis. The assays for hormones (insulin, glucagon, Pancreatic Polypeptide (PP), intestinal Glucagon-like peptide (GLP-1), and Glucose-dependent insulinotropic polypeptide (GIP)) and for cytokines (tumor necrosis factor (TNF-*α*), vascular endothelial growth factor (VEGF), interleukin-4 (IL-4), and IL-6) were conducted by a fully blinded laboratory from University of Maryland at Cytokine Core Lab using Luminex Multianalyte System.

### 2.5. Von Frey Filament Test for Diabetes-Induced Allodynia Pain

The von Frey filament test utilizes a nonnoxious stimulus to measure tactile allodynia. Behavioral testing was performed by experimenters who were blinded to the drugs being administered. The detailed method was described by Chaplan et al. [14] and Crisp et al. [15]. Mechanical paw withdrawal thresholds were measured with the up–down testing paradigm. Von Frey filaments (Cat# 58011, Stoelting, Wood Dale, IL USA) in log increments of force (2.0, 4.0, 6.0, 8.0, 10.0, 15.0, and 26 g) were applied to the mid-plantar hind paw of the rats for a duration of 1–2seconds. The stiffness of the most flexible filament that induced paw withdrawal was recorded as the withdrawal threshold. A response was considered positive when the animal's hind paw clearly lifted or flinched after being touched by the filament in at least 3 out of 5 touches. Filaments were lifted such until they bent, or the animal moved their paw in response to the touch. If an animal showed no response to any of the von Frey filaments, a value of 26 g, which represents approximately 5–10% of the animals' body weight, was recorded as stiffer filaments could raise the limb passively.

### 2.6. Paw Plantar Test for Thermal Hyperalgesia Pain of Neuropathy

The plantar test quantitatively assesses the thermal threshold for pain. The detailed method was described by Crisp et al. [15]. Rats were placed in chambers above a glass surface with surface temperature maintained at a constant 32°C (Model 336, IITC/Life Science Instruments, Woodland Hills, CA, USA). A mobile radiant heat source located under the glass is then focused onto the hind paw, and the time to paw withdrawal was recorded. The device was set to 55% heating intensity for a maximum of 10 seconds. The paw withdrawal latency was recorded by a digital timer.

### 2.7. Foot Fault Test for CNS or Muscular Deficiency

The foot fault test measures the rodent's grip strength and motor coordination skills to assess CNS or muscular effects [16]. The animals were allowed to walk on a steel wire mesh (wire diameter = 0.5 mm, 3 cm wire spacing) housed in a 38 cm × 36 cm × 17.5 cm box (W × L × D). The numbers of paw slips or misplacements are recorded manually over a 2-minute period. Healthy animals or those without neurological/muscular deficits exhibit precise placement of their feet walking over the grids while diseased animals or animals dosed with drugs that cause CNS or muscular side effects will allow their paws to slip through the metal mesh.

### 2.8. Animal Well-Being and Diabetic Progression Measurements

To help quantitate rats' well-being, three novel measures were developed and utilized in this study: a measure of fur cleanliness, a measure of fecal consistency, and a holistic measure of animal distress (animals selected for intensive supplemental care by a blinded facility's veterinarian). The development of cataracts and kidney abnormalities was also assessed.

Normally, rodents routinely groom themselves to keep their fur clean and neat. An unkempt haircoat, due to a lack of grooming, is a sign of sickness or stress in rodents. To quantitate the rodent's ability to groom, a scoring system was created for fur cleanliness. Scoring criteria were as follows: 0 = normal clean fur, 1 = a dirty tail but otherwise clean fur, 2 = a dirty tail and some brown color on the rodent's back fur, 3 = a dirty tail, some brown color on the rodent's back fur, and a wet dirty belly, and 4 = a dirty tail, a wet dirty belly, and some feces on the rodent's back fur.

Diabetic animals tend to have soft or pasty stool; therefore, fecal consistency was hypothesized to potentially be a good indicator of well-being. The following score was used to quantitate fecal consistency: 0 = full solid feces, 1 = loose feces, 2 = pasty diarrhea, and 3 = watery diarrhea.

The animals in our study were routinely assessed for signs of dehydration, sleepiness, unkempt appearance, excessive weight loss (daily body weight loss of >10%), anemia (pale eyes, ears, and paws), decreased food or water intake, skin or eye infection, and morbidity by a fully blinded veterinarian that worked for Rutgers University as the supervising veterinarian of the vivarium. Animals deemed by this veterinarian as unhealthy were given supplemental medical care or treatments such as soft gel food, subcutaneous saline injections at 5 ml/kg/day, topical antibiotics, or early sacrifice. These determinations by the veterinarian that extra care was needed were recorded as “selected for intensive supplemental care” and used as a surrogate endpoint for well-being and recorded weekly.

Cataract formation or lack thereof was noted weekly by a trained investigator following visual observations. Kidney weight and the presence or absence of kidney stones were recorded throughout the study during tissue collections.

### 2.9. Tissue Collection and Histology

Five to seven diabetic and sham rats were sacrificed at week 20 and week 25 after the first STZ injection. Pancreatic tissues were collected and preserved in 10% formalin for hematoxylin & eosin histological staining. The staining was conducted by a fully blinded contract laboratory at Pharma-Legacy following their routine procedures. After staining, the pancreases were embedded in paraffin blocks and sectioned to obtain 4–8 *μ*m thick sections. Pancreatic cells were counted under a 40x objective lens. Cell densities were expressed as cells per unit area of pancreatic islet. All remaining rats were sacrificed at week 60 (1.2 year). Kidneys were observed and weighed for abnormalities at the time of sacrifice.

### 2.10. Statistical Analysis

Sum, mean, and standard errors were calculated from each group, two-tailed (*α* = 2), student T-test statistical tests were used for comparison between treated and untreated groups at *P* < 0.05 significance level.

## 3. Results

### 3.1. Acute STZ Toxicity

In the STZ-induced rat model of diabetes, animals sometimes die within one week of STZ injection (“acute” STZ toxicity). To understand if acute STZ toxicity varied with age at the time of injection, animals in three age groups were assessed in the current study. All rats were male Sprague-Dawley with uncontrolled hyperglycemia following a STZ injection.

Group 1 animals were 6–11 weeks old with a body weight of 220–400 g at the time of STZ injection. There were sixty-seven (67) rats in group one divided into two STZ dosing regimens. Fifty (50) rats received a single dose of 65 mg/kg STZ i.v. Their average glucose level was 465 ± 26 mg/dl one week after a STZ injection, and two (2) animals had to be euthanized due to severe illness. Seventeen (17) rats received two (2) doses of 50 mg/kg i.v. 3 days apart; none of these rats died within one week of the STZ injections. The average glucose level was 133 ± 3 mg/dl three days after the first STZ-50 mg/kg dose which was comparable to the glucose level in the sham animals (112 ± 7 mg/dl). Glucose levels rose to an average of 428 ± 18 mg/dl after the second injection (5 days after the first injection and 2 days after the second injection). The total survival rate of animals in this age group (6–11 weeks old including 2 regimens) was 97% (2 of the 67 rats) throughout the study. There was also no observed difference in mortality between the smaller and larger animals in this group, body weight 220–300 g versus 301–400 g.

Group 2 animals, twenty-nine (29) rats, were 12–17 weeks old; body weight 401–500 g at the time of STZ injection, this group received a single dose of 65 mg/kg STZ i.v. Their glucose levels were 464 ± 23 mg/dl by day 5. 83% (24 of the 29 rats) died within a week of the STZ injection. Of the five surviving rats, none survived to the end of the study at week 60. One died at week 9; one at week 12; two at week 21, and one at week-25.

Group 3 animals, thirty-four (34), were 18–23 weeks old (body weight 501–600 g) at the time of STZ injection and were divided into two STZ regimens. Twenty (20) rats received a single dose of 65 mg/kg STZ i.v.; their glucose levels averaged 444 ± 6 mg/dl by day 5. Seventeen (17) of the twenty rats died from acute toxicity. Of the three surviving animals, two died 9 weeks after STZ injection, and one died 21 weeks after STZ injection. Fourteen (14) rats were dosed with 50 mg/kg STZ i.v. Despite the reduced STZ dose, all 14 rats in this age group also died within one week of the STZ injection.

No animals in the sham injection (vehicle only without STZ) died during the study period, regardless of their age at the time of injection (sixteen were 6–11 weeks old, four were 12–17 weeks old, and three were 18–23 weeks old).

Rat mortality dramatically increased in rats 12 weeks or older at the time of STZ injection. The data are summarized in Table 3.

### 3.2. Natural Course of STZ-Induced Type 1 Diabetic Model

Hyperglycemia, increased thirst (polydipsia), hyper-urination (polyuria), and glucose excretion (glycosuria), extreme hunger (polyphagia), gastric dysfunction, unexplained weight loss, and fatigue are common signs or symptoms of diabetes in humans that are observed in rats as well. To assess the natural course of the STZ-induced rat model of diabetes, these same symptoms were observed for over 60 weeks in thirty (30) diabetic rats and twenty-three (23) age-matched sham rats. All rats were maintained without antihyperglycemic treatment. Measures of fecal consistency were used to assess gastric dysfunction. Measure of fur cleanliness and other observations by a blinded veterinarian were used to assess signs of fatigue and general health conditions. Fourteen of the 30 diabetic and 13 of the 23 sham rats were sacrificed at 20 or 25 weeks after a STZ injection for histological evaluations. The remaining sixteen diabetic and ten sham rats were observed for 60 weeks after a STZ injection.

**Figure 1:**
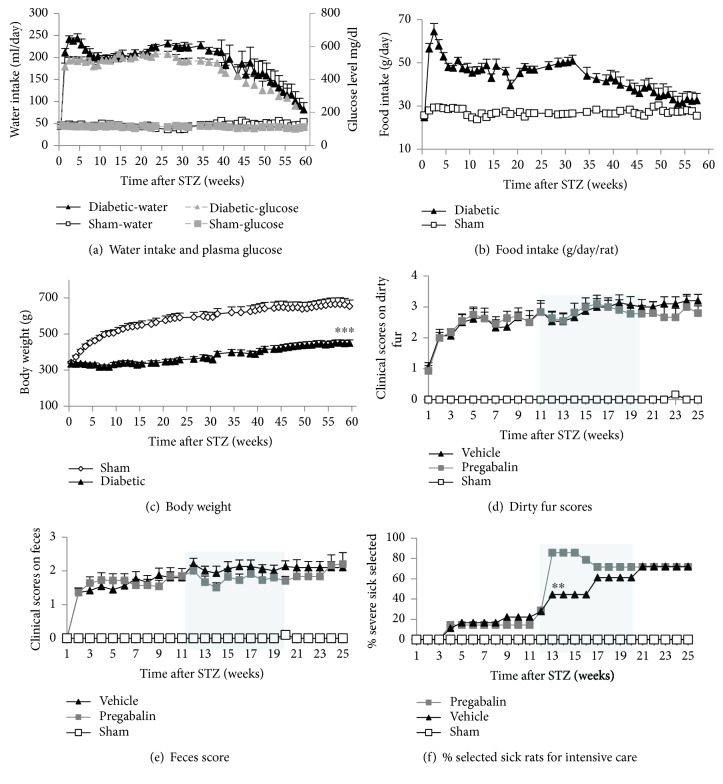
Long-term observation of hyperglycemia and general vital activities (body weight, food and water intake) and overall well-being (including fecal consistency, clean fur, and veterinarian selection of rats for intensive care or unscheduled sacrifice). Diabetic rats (*n* = 16) and age-matched sham rats (*n* = 10) were observed for 60 weeks after a STZ injection. An additional 16 diabetic rats and 13 sham animals were observed for up to 25 weeks post a STZ injection and then sacrificed for histological evaluation. Blood glucose levels were increased after a STZ injection. The hyperglycemia was stable for 40 weeks then normalized slowly to that of the sham animals (a). Water intake also increased sharply after a STZ injection, remained stable for 40 weeks, and then normalized to that of the sham animals by 60 weeks after the injection (a). Similarly, 24-hour food intake also increased after a STZ injection and remained relatively constant for at least 35 weeks before almost normalizing to that of sham rats by 60 weeks after a STZ injection (b). In comparison, body weight was significantly lower in STZ-injected animals as compared to sham controls, and this difference was maintained through the end of the study (c). In (d), (e), and (f), a separate group of 12 diabetic rats was treated with Pregabalin which was administrated in their drinking water ad libitum for 8 weeks from 12 to 20 weeks after a STZ injection (shadow area). Pregabalin was dosed at 30 mg/kg/day on day 1 of week 12 then at 15 mg/kg through week 18 then at 20 mg/kg/day through week 20. The diabetic rats had poorer quality haircoats and less well-formed feces than normal sham rats throughout the 25 weeks of quantitation. Treatment with Pregabalin had no effect on the diabetic rats' ability to maintain their fur clean or the consistency of their feces (d–e). The percent of rats in each group selected by a veterinarian for intensive supplemental care or unscheduled sacrifice increased with time in the STZ-injected rats (f). Pregabalin treatment further exacerbated the number of animals selected for intensive supplemental or unscheduled sacrifice (f). *n* = 10–32, percent, mean ± standard errors (s.e.) were calculated from each group at each time point, two-tailed (*α* = 2), Student's *t*-test, ^∗∗^*p* < 0.01 (diabetic group treated with Pregabalin compared to diabetic group treated with vehicle), ^∗∗∗^*p* < 0.001 (diabetic rats compared to normal sham rats).

Blood glucose was monitored weekly or biweekly. Hyperglycemia was confirmed within a week of STZ injection (average of 465 ± 26 mg/dl). The sham rats showed normal glucose levels throughout the study (113 ± 2 mg/dl). The percent HbA1C was measured at 11 and 20 weeks after a STZ injection to assess glycated hemoglobin, a surrogate for average blood glucose level over the previous 2–3 months. Both blood glucose levels (Figure 1(a)) and %HbA1C were significantly increased after a STZ injection. %HbA1C levels were 10.8 ± 0.4 and 10.6 ± 0.22 in STZ-induced diabetic rats as compared to 4.7 ± 0.1 and 4.5 ± 0.1 in sham rats at 11 and 20 weeks post a STZ injection, respectively. Surprisingly, the glucose levels in diabetic rats tended to normalize naturally starting 40 weeks after a STZ injection (Figure 1(a)). Likewise, food and water intake of the diabetic rats rose rapidly after a STZ injection then remained relatively constant for 30 to 40 weeks, before almost normalizing to that of normal sham rats 60 weeks after a STZ injection (Figures 1(a) and 1(b)). The normalization kinetics of food and water intake and plasma glucose levels appear similar. All animals were sacrificed at week 60.

The body weight in sham rats increased with age as expected. Diabetic rats showed less body weight gain than sham animals (Figure 1(c)), particularly in the first 10–15 weeks after a STZ injection. Polyuria was subjectively observed since the cages of the diabetic rats required bedding changes every day compared to weekly bedding changes required for the cages of sham rats (2 rats/cage). Unlike food and water consumption and glucose levels, the difference in body weight between sham and STZ-induced diabetic rats was maintained throughout the study.

The rats' ability to maintain a clean haircoat and the consistency of their feces were used as surrogate endpoints of both gastric dysfunction and the animals' well-being. The diabetic rats had much poorer quality haircoat and less well-formed feces than normal sham rats throughout the 25-week measurement period, and although not quantitated beyond 25 weeks, these measures appeared to normalize with glucose level after 40 weeks. Fur cleanliness scores are plotted in Figure 1(d), and fecal consistency is plotted in Figure 1(e). Treatment with Pregabalin, for 8 weeks starting at week 12, had no effect on the diabetic rats' ability to maintain their coats or the consistency of their feces (Figures 1(d) and 1(e)).

Diabetic rats that demonstrated adverse clinic signs over the course of the study were assessed by the animal facility's veterinarian who determined whether supplemental care or humane euthanasia was indicated. The veterinarian's identification of rats in need of supplemental care or humane euthanasia is plotted in Figure 1(e) for the first 25 weeks after a STZ injection and appears to be a good surrogate for well-being. There were 15 rats in the sham group, 18 control diabetic rats who received no treatment, and 12 diabetic rats who received Pregabalin dissolved in their drinking water from weeks 12 to 20 after the STZ injection. Pregabalin was dosed at 30 mg/kg/day on day 1 then at 15 mg/kg from day 2 of week 12 through week 18 then at 20 mg/kg/day through week 20. The Pregabalin dose was adjusted due to severe side effects at 30 mg/kg/day and due to a decrease in efficacy after several weeks at 15 mg/kg/day. None of the sham rats was selected for supplemental care while there was an increase in the number of rats selected for supplemental care in the control diabetic group and an even larger increase in the diabetic group treated with Pregabalin presumably due to Pregabalin's side effects (Figure 1(f)) over the course of the study.

### 3.3. Assessment of Diabetic Complications

Long-term uncontrolled diabetics usually develop multiple complications; the most common are cardiovascular disease (where hyperlipidemia is a biomarker), eye damage particularly in younger patients such as cataracts, kidney damage (nephropathy), and nerve damage (neuropathy).

#### 3.3.1. Hyperlipidemia

A blood lipid profile including total cholesterol (TC) and triglycerides (TG) were tested at weeks 11, 15, 19, and 23 after a STZ injection. Both total cholesterol and triglyceride levels were significantly increased in STZ-injected diabetic rats compared to normal sham rats as would be expected in diabetic animals. Total cholesterol and triglyceride levels were generally constant with an average of 123 ± 8 mg/dl (TC) and 526 ± 12 mg/dl (TG) in diabetic rats (*n* = 15) and an average of 106 ± 5 mg/dl (TC) and 142 ± 9 mg/dl (TG) in sham rats (*n* = 10), mean ± s.e., *p* < 0.01, *a* = 2, Student's *t*-test compared between diabetic rats and normal sham rats. Due to the difficulty in collecting blood samples from diabetic rats, lipid levels were not monitored after week 23.

#### 3.3.2. Diabetic Nerve Damage: Neuropathic Pain

**Figure 2:**
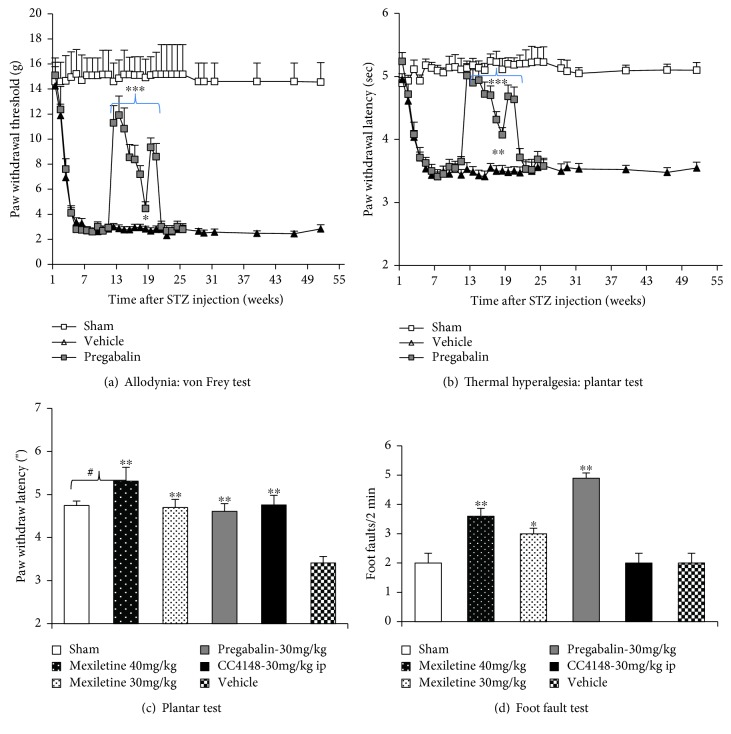
Effect of repeated dosing Pregabalin or single dosing Mexiletine, Pregabalin, and CC4148 on neuropathic pain measures and coordination test in STZ-induced diabetic rats. Neuropathic pain was assessed every 1–2 weeks with the von Frey test for tactile allodynia pain (a) and the plantar test for thermal hyperalgesia pain (b) in STZ-injected or sham-injected animals. Neuropathic pain fully developed over 5 weeks after a STZ injection and lasted through the end of the study, more than one year. Chronic administration of Pregabalin in the animals' drinking water for 8 weeks inhibited pain (a–b). However, Pregabalin showed side effects at 30 mg/kg/day, so the dose was reduced to 15 mg/kg/day on day 2 of week 12. After 3–4 weeks of dosing, the efficacy of Pregabalin started to attenuate, so the dose was increased to 20 mg/kg/day for the final two weeks of dosing. A single dose of Pregabalin 30 mg/kg by oral gavage also caused side effects of sleepiness and a decrease in coordination as measured by the foot fault test (d). An acute administration of Mexiletine at 30 and 40 mg/kg by oral gavage significantly and dose-responsively inhibited thermal hyperalgesia via the plantar test (c) with side effects showed in the foot fault test (d). A selective Nav1.7 blocker, CC4148, at 30 mg/kg by an intraperitoneal injection (i.p.) showed significant inhibition on thermal hyperalgesia pain (c) and no evidence of central nerve system and muscular adverse effects via the foot fault test (d). Behavior tests were conducted 30′ after oral gavage or i.p. and between 9 and 10 AM in the chronic study. *N* = 10–12, mean ± s.e. were calculated from each group at each time point, two-tailed (*α* = 2), Student's *t*-test, ^∗^*p* < 0.05, ^∗∗^*p* < 0.01, ^∗∗∗^*p* < 0.001 (compared to vehicle), #*p* < 0.05 (compared to sham).

The development of neuropathic pain after a STZ injection was assessed with the von Frey filament test for tactile allodynia (Figure 2(a)) and the plantar test for thermal hyperalgesia (Figure 2(b)). Neuropathic pain slowly develops over the first 5 weeks after a STZ injection and remained at consistent levels throughout the study (60 weeks). Pain did not resolve with the resolution of hyperglycemia. Twelve rats were dosed repeatedly with Pregabalin from 12–20 weeks after a STZ injection (Figures 2(a) and 2(b)). Pregabalin was administrated in the animals' drinking water ad libitum (see Table 2). Pregabalin was dosed at 30 mg/kg/day on day 1 of week 12 then at 15 mg/kg from day 2 through week 18. Pregabalin was dosed at 20 mg/kg/day for the final two weeks, 18–20 (Figures 2(a) and 2(b)). The rats dosed chronically with Pregabalin were sacrificed at week 25 after a STZ injection. Pregabalin inhibited both allodynia (Figure 2(a)) and thermal hyperalgesia (Figure 2(b)) with tachyphylaxis (efficacy declining with time). Pregabalin-treated animals displayed significant side effects (sleepiness, loss of coordination in the foot fault test, Figure 2(d)).

Ten sham rats and 73 STZ-injected diabetic animals were tested for neuropathic pain. Of these 73 diabetic rats, 32 rats received single doses of Mexiletine (30 or 40 mg/kg p.o.), Pregabalin (30 mg/kg p.o.), or a peripherally restricted, selective Nav1.7 blocker, CC4148 (30 mg/kg i.p.) 5–6 weeks after a STZ injection and were assessed for thermal hyperalgesia with the plantar test (Figure 2(c)). All three drugs inhibited thermal hyperalgesia (Figure 2(c)), but Mexiletine and Pregabalin caused an increase in foot faults (Figure 2(d)).

### 3.4. Other Common Diabetic Organ Pathologies

#### 3.4.1. Ocular Pathology

**Figure 3:**
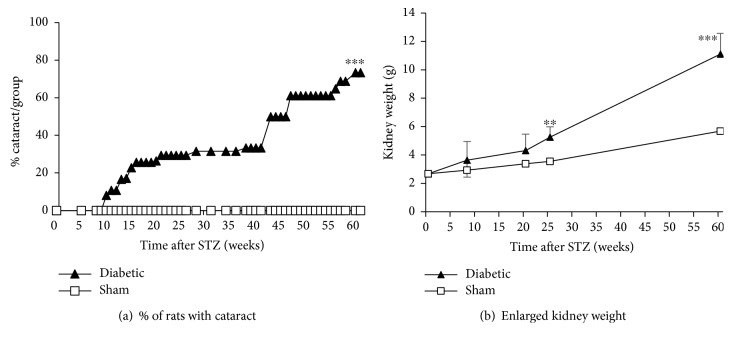
Long-term observations for cataract formation and kidney enlargement in STZ- and sham-injected rats. The presence of cataracts was checked by visual inspection weekly or biweekly. The first cataracts were observed 9 weeks after a STZ injection, and 73% of the animals had cataracts 60 weeks after a STZ injection. No cataracts were observed in age-matched sham animals (a). Five to 6 STZ-injected animals died or were sacrificed at weeks 9, 21, and 25; in these animals, their kidneys were enlarged compared to age-matched sham animals. At terminal sacrifice, week 60, all STZ-injected animals (*n* = 15) had some degree of kidney enlargement and kidney stones, sham rats did not (*n* = 5–10) (b). *N* = 5–20, percent, mean ± s.e. were calculated from each group at each time point, *α* = 2, Student's *t*-test, ^∗∗^*p* < 0.01, ^∗∗∗^*p* < 0.001 (compared to sham).

Many of the rats developed cataracts in the diabetic group. Cataracts were observed as early as 9 weeks after a STZ injection. The number of STZ-injected animals with cataracts continued to increase throughout the study period, even as the hyperglycemia resolved. At the end of the study, 60 weeks after a STZ injection, 73% of the diabetic rats had cataracts. No cataracts were found in age-matched sham rats (Figure 3(a)).

#### 3.4.2. Kidney Pathology

As animals died or were sacrificed, their kidneys were checked for stones and measured for weights. All STZ-injected rats had some degree of kidney enlargement and kidney stones at the time of final sacrifice, 60 weeks after a STZ injection. Although only a few kidneys were examined at weeks 9, 21, and 25 after a STZ injection (*n* = 5–6), an increase in kidney weight (including sand-like stones) in the diabetic animals as compared to their normal sham controls was observed at all time points, reaching statistical significance at week 25 and week 60 after a STZ injection (Figure 3(b)). No kidney stones were found in sham animals.

### 3.5. Metabolic Hormone Plasma Levels after a STZ Injection

**Figure 4:**
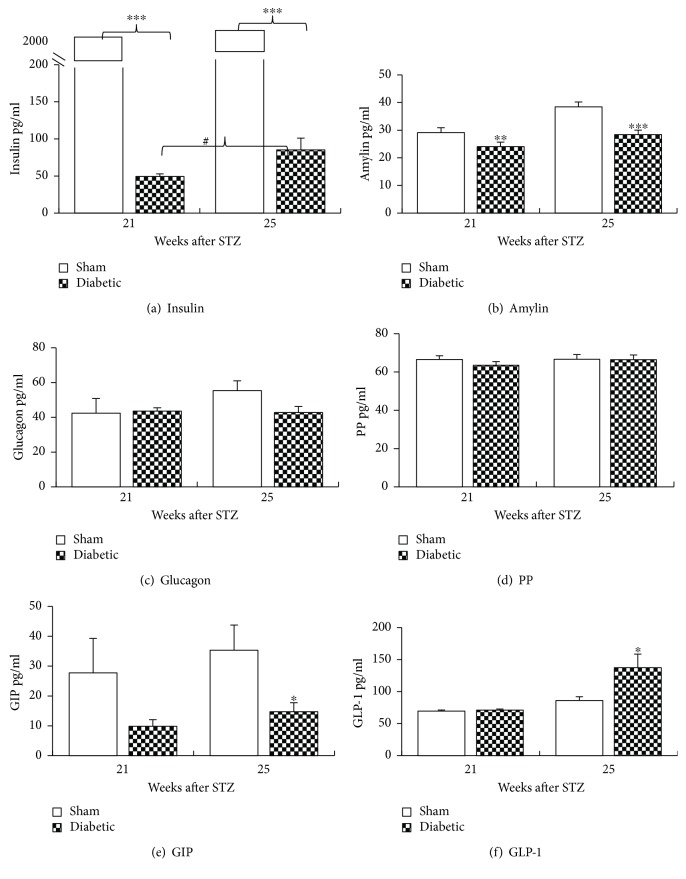
Plasma levels of metabolic hormones after a STZ injection. Plasma was collected at week 21and week 25 after a STZ injection under nonfasting condition for the quantification of hormone levels. STZ challenge significantly reduced insulin (a) and amylin (b) levels compared to sham rats. The insulin level improved slightly between weeks 21 and 25 (#*p* < 0.05) in STZ-injected rats. STZ injection did not show significant effect on glucagon (c) and PP levels (d). GIP decreased in diabetic animals although statistical significance was only reached at week 25 and not at week 21 may be due to small sample numbers (e). GLP-1 increased in diabetic animals at week 25 but not week 21 (f). *n* = 3–5 at week 21, and *n* = 6–10 at week 25, mean ± s.e. Student's *t*-test, *a* = 2, ^∗^*p* < 0.05, ^∗∗^*p* < 0.01, ^∗∗∗^*p* < 0.001 (diabetic rats compared to normal sham rats).

To further characterize the STZ-induced diabetic model, plasma cytokine and metabolic hormone levels were measured, and histological studies were run at 21 and 25 weeks after a STZ injection. STZ injection significantly reduced insulin and amylin levels compared to normal sham rats (Figures 4(a) and 4(b)) without causing a significant difference in glucagon or PP levels (Figures 4(c) and 4(d)). Interestingly, the insulin levels statistically significantly increased in untreated diabetic rats between weeks 21 and 25. STZ-induced diabetes appeared to decrease GIP levels compared to sham, but the data only reached significance at week 25; data were collected from 3 to 5 animals at week 21 and 6–10 animals at week 25 (Figure 4(e)). An increase in GLP-1 levels was seen at 25 but not 21 weeks after a STZ injection (Figure 4(f)).

### 3.6. Plasma Levels of Inflammatory Cytokines after a STZ Injection

**Figure 5:**
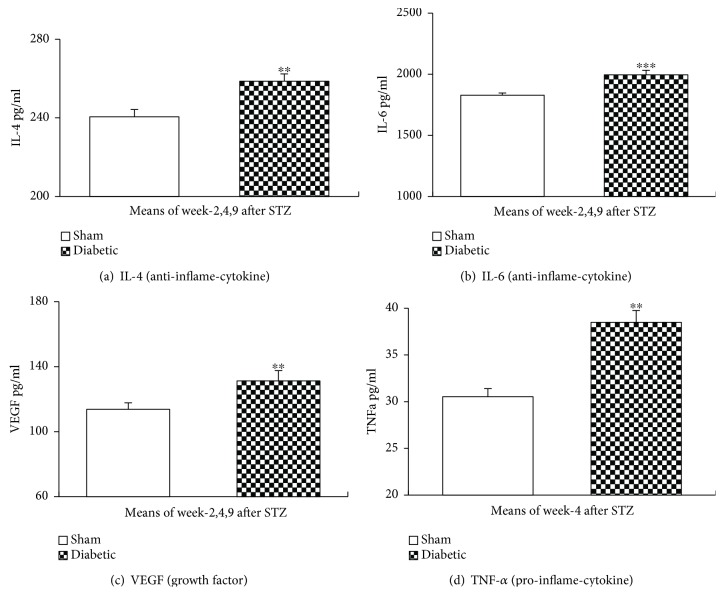
Plasma levels of inflammatory cytokines after a STZ injection. Plasma was collected 2, 4, and 9 weeks after a STZ injection to measure inflammatory cytokines. STZ challenge significantly increased IL-4 (a), IL-6 (b), VEGF (c), and TNF-*α* ((d), week 4 only) levels in diabetic rats without antidiabetic treatment. Mean ± s.e. Student's *t*-test, *a* = 2, *n* = 5–6/diabetic group, *n* = 3–6/sham group, ^∗∗^*p* < 0.01, ^∗∗∗^*p* < 0.001 (compared to sham group).

Plasma was collected from both diabetic and sham rats at weeks 2, 4, and 9 after a STZ or sham injection to measure inflammatory cytokines of IL-4, IL-6, and VEGF and at week 4 for TNF-*α*, (*n* = 5–6 for diabetic rats, *n* = 3–6 for sham rats/week). No significant differences were seen between sampling weeks, so samples from the different weeks were averaged. STZ-injected diabetic rats had significantly increased IL-4, IL-6, VEGF, and TNF-*α* as compared to sham rats (Figures 5(a)–5(d)).

### 3.7. Histological Tissue Staining for Pancreatic Islet *β*-cell Density and Inflammatory Cell Infiltration

**Figure 6:**
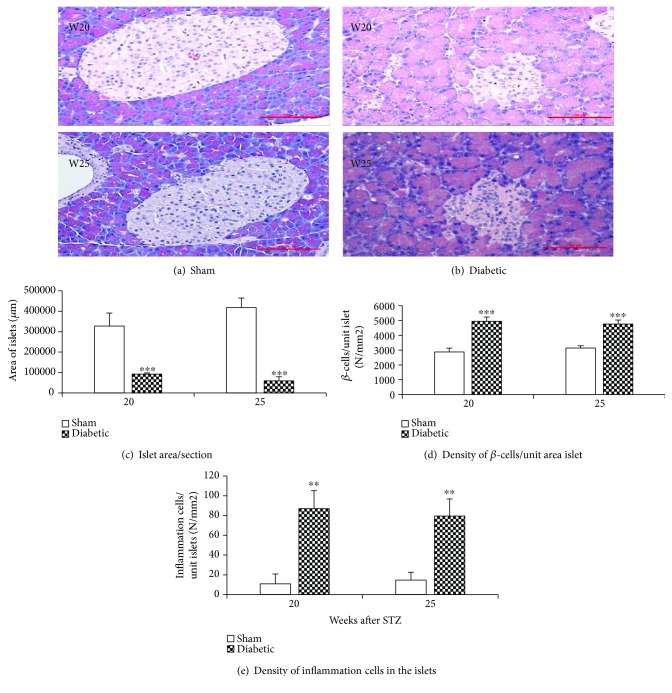
(a–e) Islet area, *β*-cell density, and inflammatory cell infiltration into pancreatic islets. Representative images of the pancreas from rats 20 and 25 weeks after STZ or sham injection stained with hematoxylin & eosin stain (a–b). The pancreatic islet area and *β*-cell and inflammatory cell density in each islet were counted under a 40x objective lens. The total area of pancreatic islets was reduced after a STZ injection compared to that in age-matched sham-injected animals (c); however, the *β*-cell density was significantly increased in diabetic rats compared to sham animals (d). More inflammatory cells infiltrated into pancreatic islets in diabetic rats (e). *n* = 5–6/group, mean ± s.e. Student's *t*-test, *a* = 2, ^∗∗^*p* < 0.01, ^∗∗∗^*p* < 0.001 (compared to age-matched sham group).

To evaluate pancreatic pathology after a STZ injection, histological samples were collected from both STZ-injected diabetic and normal sham rats at weeks 20 and 25 after the injection. Pancreatic tissue was stained with hematoxylin & eosin stain (H&E stain). The area of the pancreatic islets was measured, and the *β*-cell and inflammatory cell density in each islet were counted under a 40x objective lens and normalized per unit islet area. Representative images from sham and STZ-injected animals at week 20 and week 25 post injection are shown in Figures 6(a) and 6(b). As expected, the total area of pancreatic islets/section was significantly reduced in diabetic rats compared to sham rats (Figure 6(c)); however, the *β*-cell density was significantly increased in diabetic rats compared to sham rats (Figure 6(d)). The STZ-injected diabetic rats also had significantly more inflammatory cells (mostly T-cells) infiltrating into pancreatic islets as compared to sham rats (Figure 6(e)).

## 4. Discussion

Diabetes is induced with many different STZ dosing regimens in rodents [2, 4–6, 13]. With rats, a single dose of 65 mg/kg STZ i.p. or i.v. is used most frequently [5, 6]. We used either a single 50 or 65 mg/kg dose or two 50 mg/kg doses of STZ by i.v. injection in rats. We experienced surprisingly high mortality rates in our study regardless of dosing regimens that was tightly correlated with age. Most rats older than 12 weeks at the time of STZ injection died within 5 days of dosing (82.8% age 12–18 weeks and 91.2% older than 18 weeks), and all died by 25 weeks after a STZ injection. Only 3% of younger rats, age 6–11 weeks old, died within one week of a STZ injection, and no additional rats died during the study period. Also, we did not see a correlation between mortality and age within 6–11 weeks old rats. The sharp increase in mortality of older rats was striking considering the average 2–2.5 years (24–30 months) life-span of normal male Sprague-Dawley rats in the laboratory. STZ has documented acute toxic effects on multiple organs [17, 18], and it seems likely that STZ toxicity rather than diabetic complications contributed to this acute mortality given that animals who survived the first week generally lived for several more months; however, the reason for this age dependence is not obvious. A review by Andreollo et al. stated: a rat at age of 3, 6, 12, 18, 24, or 30 months is estimated equivalently to human 12 (Puberty), 18, 30, 45 (menopause), 60 or 75 years old, respectively [19, 20]. The mechanism of the sharp increase in STZ-induced mortality could not be explained with an age-related decline in physical condition considering rat age at 12 weeks or older are still young adults. Interestingly, oxaliplatin, a chemotherapy did not show this age-dependent toxicity (internal data, not included here). The mechanism of acute age dependence of STZ toxicity remains unknown.

Common symptoms in diabetic patients include polyuria, polydipsia, polyphagia, and fatigue. Rats with STZ-induced diabetes shared these symptoms with measurably increased food and water intake, increased urination, and diarrhea suggesting intestinal dysfunction and reduced general activity suggesting fatigue starting a few weeks after a STZ injection and continuing until model recovery. Diabetes is a chronic debilitating condition. To monitor diabetic animals for more than a year, we developed noninvasive, low stress, reproducible assessments scoring their ability to maintain a clean haircoat (a sign of fatigue), the consistency of their feces (sign of gastric-intestinal function), and a holistic measure of animal distress (general illness) that proved to be a sensitive measure of model recovery.

The natural recovery of this model was consistent with reports from other researchers who described three glycemic phases: phase 1 where hyperglycemia was maintained (1 to 36–40 weeks after a STZ injection), phase 2 natural recovery (40 to 90 weeks after a STZ injection), and phase 3 normoglycemia maintained (90 to 120 weeks after a STZ injection) [21]. Glucose levels in diabetic rats normalized starting at week 36 and almost reach full recovery at week 60. HbA1c levels were equivalent at weeks 11 and 20 confirming the model's stability.

Pancreatic histology revealed a decrease in the size of pancreatic islets, a significant increase in the *β*-cell density per area of islet, and an increase in inflammatory cell infiltration in the pancreatic islets in diabetic rats compared to the age-matched normal rats. The increase in *β*-cell density suggests a compensatory response to the STZ damage. Recent work with pancreatic cells has shown that it is possible to convert rodent non-*β*-cells to *β*-cells *in vivo* and ex vivo and to direct differentiation of human pluripotent stem cells into *β*-cells [22, 23]; further work would be needed to prove that this hypothesis translates to humans.

Inflammation plays an important role in diabetes mellitus and its complications [7–12, 24, 25]. The pathogenesis in type 1 and the late phase of type 2 diabetes mellitus is associated with T-cell infiltration of the pancreatic islets (insulitis) and is characterized by a progressive T-cell-mediated destruction of the insulin-producing *β-*cells [7, 10]. IL-4, an anti-inflammatory cytokine, and TNF-*α*, a proinflammatory cytokine, levels shift in both type 1 and type 2 diabetes [10, 12, 25]. Expression of IL-4 prevented the onset of insulin-dependent diabetes mellitus in nonobese diabetic mice [10, 11]. The observed increase in inflammatory cells (mostly T-cells) infiltration and changes in cytokines levels confirm inflammatory pathology in diabetes [7–12, 25].

Diabetic complications involve almost all organs; hyperlipidemia and other cardiac damage, cataracts and retinal damage, nephropathy, and neuropathy [12, 25–27] are major sources of morbidity in poorly controlled diabetic patients.

Neuropathy has no known cure and remains an important unmet medical need [1, 23, 24, 26]. In our model, neuropathic pain gradually developed over 5 weeks and continued throughout the study (>1 year) even as the diabetes itself resolved suggesting that the neuropathy is due to long-term neuronal damage that is not readily reversible. Pregabalin, an approved analgesic, was efficacious throughout the treatment although tachyphylaxis was observed. Unfortunately, Pregabalin adversely affected general well-being and impaired coordination suggesting central and/or neuromuscular-driven side effects as well. Pregabalin's ability to reverse pain but not these other measures of well-being suggests that unkempt appearance in diabetic rats is more related to their fatigue than pain. Diabetes mellitus and its neuropathic pain are frequently associated with inflammation and upregulated expression of NaV1.7 [26, 28]. Oral administration of a nonselective sodium channel inhibitor, Mexiletine, significantly and dose-responsively inhibited pain but caused neuromuscular side effects and increased sleepiness. A highly selective NaV1.7 blocker, CC4148, blocked pain with no evidence of CNS or neuromuscular side effects, suggesting that selective inhibition of NaV1.7 is a promising pain target.

Cataract formation, kidney damage, and dyslipidemia, common morbidities in diabetic patients [25, 27], were recapitulated in this diabetic rat model. Cataracts were found only in diabetic rats suggesting that cataract formation is related to diabetes rather than age. Kidney enlargement and kidney stones were also observed only in diabetic rats with all animals having both by 60 weeks after a STZ injection. As in diabetic patients, dyslipidemia was found in diabetic rats with both cholesterol and triglyceride levels significantly increased.

## 5. Conclusions

There is a strong age-dependent toxicity associated with the STZ injection that makes this diabetic model unreliable. To reduce mortality, rats 12 weeks or younger at the time of STZ injection should be chosen or a significant reduction in STZ dose explored if older animals are needed. Fecal consistency and fur cleanliness scores provide novel endpoints for monitoring rodent behaviors in chronic diabetic studies, which are noninvasive, stress-free, repeatable, and precise measures of the animal's health. The observed increased *β*-cell density suggests pancreatic regeneration and may explain the mechanism of natural recovery in STZ-induced diabetic models. The observed inflammatory endpoints suggest factors that could be exploited for future anti-inflammatory diabetic treatment development. Overall, the STZ-induced model of diabetes recapitulates human diabetes and can facilitate new drug development for the complications of diabetes.

## Figures and Tables

**Table 1:** Animal groups used to assess age-dependent STZ toxicities.

Groups	Group 1 (BW 220–400 g)	Group 2 (BW 401–500 g)	Group 3 (BW 501–600 g)	Total
Age (weeks)	6–11	12–17	18–23	
Number of rats receiving single STZ 65 mg/kg i.v.	50	29	20	99
Number of rats receiving single STZ 50 mg/kg i.v.	0	0	14	14
Number of rats receiving two STZ 50 mg/kg i.v. 3 days apart	17	0	0	17
Total rats injected with STZ	67	29	34	130
Total sham-injected rats	16	4	3	23

**Table 2:** Treatment groups of diabetic neuropathic pain.

Group number	Group name	Compound name	Doses (mg/kg)	Route	Volume (ml/kg)	Animal numbers
One-time acute treatment						
1	Sham	N/A	N/A	N/A	N/A	10
2	20% PEG600/water	Vehicle	0	i.p.	2	10
3	Mexiletine 30 mg/kg	Mexiletine	30	p.o. gavage	2	10
4	Mexiletine 40 mg/kg	Mexiletine	40	p.o. gavage	2	10^a^
5	Nav1.7 blocker 30 mg/kg	CC4148	30	i.p.	2	10
6	Pregabalin 30 mg/kg	Pregabalin	30	p.o. gavage	2	10
Chronic treatment for 8 weeks						
7	Sham	N/A	N/A	N/A	N/A	15^b^
8	Water	Vehicle	0	p.o. drinking	Ad libitum	18
9	Pregabalin 30/15/20 mg/kg	Pregabalin	30/15/20^c^	p.o. drinking	Ad libitum	12

^a^Mexiletine 40 mg/kg group of rats had been dosed with Mexiletine 30 mg/kg one week prior, during week 5 to week 6 after STZ injection. ^b^Ten of the sham rats were reused from the sham group in the acute study. ^c^Pregabalin was dosed daily for 8 weeks from week 12 to week 20. Pregabalin was given at 30 mg/kg/day on day 1 and reduced to 15 mg/kg/day from day 2 onwards due to adverse side effects. After 6 weeks of treatment, the dose was increased to 20 mg/kg/day for the last two weeks of the study due to tachyphylaxis (efficacy declined with time).

**Table 3:** STZ-induced mortality appears to depend on the age of the rat at the time of STZ injection.

Body weight (g)	220–400	220–400	401–500	501–600	501–600	Total
Age (weeks)	6–11	6–11	12–17	18–23	18–23	
Rats received single STZ 65 mg/kg i.v.	50	0	29	20	0	99
Rats received single STZ 50 mg/kg i.v.	0	0	0	0	14	14
Rats received double STZ 50 mg/kg i.v. 3 days apart	0	17	0	0	0	17
Total rats injected with STZ	50	17	29	20	14	130
Rats surviving the 1^st^ week after STZ i.v.	48	17	5	3	0	73
% surviving the 1^st^ week post injection	96%	100%	17.2%	8.8%	0%	—
Week of mortality after the 1^st^ week	—	—	9, 12, 21, 21, 25	9, 9, 21	—	—
% surviving to end of study at week 60	96%	100%	0%	0%	0%	—

## Data Availability

Regarding the data availability, all data used to support the findings of this study are included within the article.

## Abbreviations

STZ: Streptozotocin
NaV: Voltage-gated sodium channel
H&E: Hematoxylin & eosin stain
i.v. or i.p.: Intravenous injection or intraperitoneal injection
IL-4: Interleukin 4
TNF-*α*: Tumor necrosis factor-*α*
VEGF: Vascular endothelial growth factor
PEG: Polyethylene glycol
HbA1C: Glycated hemoglobin
TC: Total cholesterol
TG: Triglyceride
PP: Pancreatic polypeptide
GLP-1: Glucagon-like peptide-1
GIP: Glucose-dependent insulinotropic polypeptide
GABA: Gamma-aminobutyric acid.
